# Sequential extraction of value-added bioproducts from three *Chlorella* strains using a drying-based combined disruption technique

**DOI:** 10.1186/s40643-023-00664-1

**Published:** 2023-07-22

**Authors:** Zahra Izanlou, Mahmood Akhavan Mahdavi, Reza Gheshlaghi, Arash Karimian

**Affiliations:** Department of Chemical Engineering, Ferdowsi University of Mashhad, Azadi Square, Pardis Campus, 91779-48944, Mashhad, Iran

**Keywords:** *Chlorella*, Bioproducts, Disruption, Drying, Sequential extraction, Biorefinery

## Abstract

In this study, the sequential extraction of the three types of biochemicals from microalgae is employed, which is a more realistic and practical solution for large-scale extraction of bioproducts. The drying, grinding, organic solvent treatment, and ultra-sonication were combined to disrupt cells and sequentially extract bioproducts from three microalgae strains, *Chlorella sorokiniana* IG-W-96, *Chlorella* sp. PG-96, and *Chlorella vulgaris* IG-R-96. As the drying is the most energy-intensive step in cell disruption and sequential extraction, the effect of this step on sequential extraction deeply explored. The results show that total ash-plus contents of biochemicals in freeze-dried samples (95.4 ± 2.8%, 89.3 ± 3.9%, and 77.5 ± 4.2 respectively) are higher than those in oven-dried samples (91.0 ± 2.8%, 89.5 ± 3.0%, 71.4 ± 4.8%, respectively) showing the superiority of freeze drying over oven drying merely for *Chlorella vulgaris* IG-R-96 (p-value = 0.003) and non-significant variation for *Chlorella sorokiniana* IG-W-96 (p-value = 0.085) and *Chlorella* sp. PG-96 (p-value = 0.466). Variation among biochemical contents of strains is due to the difference in cell wall strength confirmed by TEM imaging. The freeze-dried samples achieved higher lipid yields than oven-dried samples. The total carbohydrate yields followed the same pattern. The extraction yields of total protein were higher in freeze-dried samples than in oven-dried. Total mass balance revealed that drying-based sequential extraction of value-added bioproducts could better demonstrate the economic potential of sustainable and renewable algal feedstock than independent assays for each biochemical.

**Graphical Abstract:**

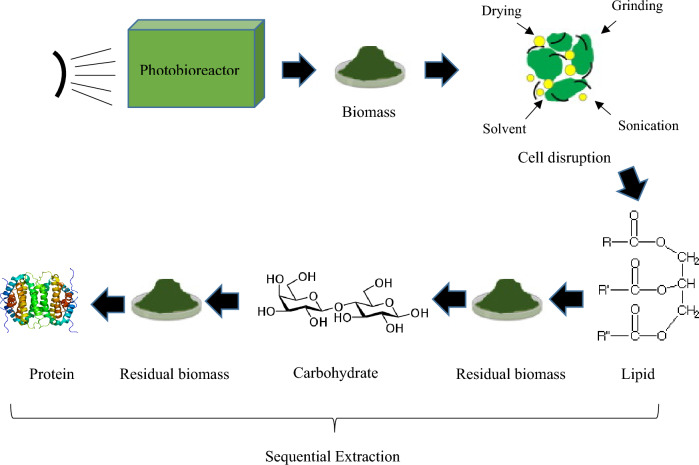

## Introduction

Microalgae feedstock is a renewable source for producing commodity bioproducts such as carbohydrates, proteins, lipids, and pigments. These biomacromolecules are used in a variety of applications. The carbohydrates are used in the production of biopolymers and ethanol. Microalgae proteins are low-volume and valuable substances in the food and pharmaceutical industries. The lipids can be used in biodiesel production, cosmetics, and nutraceutical industries.

There are several traditional (Santos et al. 2015) and modern (Kim et al. 2015) methods available for the measurement and extraction of bioproducts from microalgae. Nevertheless, there is no robust treatment protocol suitable for potential use at industrial level. The early treatments include independent assays for each type of macromolecule, but these approaches do not cover the extraction effect of one component on the other ones. A significant consideration in the sequential extraction of all valuable products from algae is the effect of extracting one component on the individual yields of the remaining products. The *Dunaliella tertiolecta* biomass sample consisted of 22.0 wt% lipid, 27.2 wt% protein, and 40.5 wt% carbohydrate using independent assays. After lipid extraction, the residual biomass contained 35.0 wt % protein and 51.9 wt % carbohydrate (Kim et al. 2015). It was found that the highest amount of protein (58%) and carbohydrate (20%) for *Scenedesmus obliquus* was obtained from lipid-extracted algae (LEA), concluding that the initial extraction of lipid increased the extraction efficiency of carbohydrates and proteins (Ansari et al. 2015) compared to commonly reported 16–32% protein and 45–50% carbohydrate contents in *Scenedesmus obtusiusculus*; respectively (Schulze et al. 2016). In another study, the lipid and carbohydrate contents of *Chlorella vulgaris* biomass were 18.1% and 40.2%, respectively. However, after lipid extraction, the total carbohydrate in the microalgae biomass was slightly reduced (37.3%) (Lam et al. 2014). Another study reported that before the extraction of lipids, the carbohydrates and protein contents of *Chlorella* sp. KR-1 accounted for 36.1% and 16.6% of the microalgae biomass, respectively. The carbohydrate and protein contents increased to 49.7% and 28.5% in the lipid-extracted residual biomass (Lee et al. 2015). Thus, to this end, a unified method for simultaneous assay of biochemical composition in a single microalgae sample was presented (Chen and Vaidyanathan 2013). It was concluded that sequential extraction of components through the unified procedure was economically favorable so that it saved sample (by 79%), time (67%), chemicals (34%), and energy (58%) when compared to the corresponding assay for each component, carried out individually on different samples.

Increasing the extraction yield of carbohydrates, proteins, and lipids in a sequential process highly depends on the success of the cell disruption procedure. Generally, there are mechanical and non-mechanical methods that are used for cell disruption. These methods are reviewed elsewhere (Günerken et al. 2015). Drying seems to be more appealing since, in addition to the destruction or deformation of the cell wall and safe subsequent extraction of triacylglycerols, it reduces the cost of handling, as well as transportation and packaging. However, some of these methods are not economically sustainable (Pohndorf et al. 2016). So, the choice of drying technique influences the scale of microalgae extraction. Freeze drying is an expensive method, especially for large scale, but it eases lipid extraction. The intracellular materials are hard to extract from biomass with an organic solvent treatment without disrupting the cell wall, but extraction from freeze-dried biomass is relatively easy. The oven drying provided the highest yields of all products, followed by freeze-drying, while sun drying significantly lowered extraction yields (Ansari et al. 2015).

It has been demonstrated that a combination of disruption techniques could be more effective (Phong et al. 2018). The highest lipid and fatty acids productivity, 11.9% of dry weight, was obtained by the osmotic shock method combined with chloroform:methanol (1:1 v/v) organic solvent treatment (El-Sheekh and Hamouda 2016). Physical, chemical, and enzymatic pretreatments were applied to three microalgal species. The results confirmed that the combination of acid pretreatment and enzymatic hydrolysis enhanced the breakdown of complex carbohydrates into simple sugars in the bioethanol production process from microalgal biomass (Hernández et al. 2015). Under the optimized combination of four disruption methods, 72.4% of the protein was extracted from the microalgae *Chlorella pyrenoidosa* (Zhang et al. 2018). Different organic solvents were used for dry and wet biomass to study lipid yield and their effects on other biochemicals. The isopropanol/hexane (2:1 v/v) and dichloromethane/methanol (2:1 v/v) were found to be appropriate organic solvents for wet and dry biomass, respectively (Ansari et al. 2017).

In this study, the novel sequential extraction of bioproducts (lipid, carbohydrate, and protein) from the same sample is introduced. The extraction approach combines four disruption methods, including drying, grinding, organic solvent treatment, and ultrasonication. This study also determines the best drying method from an economic point of view. This methodology was applied to three different algal strains further to include the effect of species on extraction yield. As drying of algal cells may variably affect cell walls and subsequent cell wall disruption, both oven drying and freeze-drying were tested to evaluate the most effective drying method in a sequential extraction of bioproducts.

## Materials and methods 

### Algal strains

Three microalgae species used in this study. *Chlorella vulgaris* IG-R-96 (GenBank accession no: MF459966), *Chlorella sorokiniana* IG-W-96 (GenBank accession no: MF459965) and *Chlorella* sp PG-96 (GenBank accession no: MG437300), were isolated from local wastewater facilities and identified based on 18S rRNA sequencing.

### Microalgae solution

A sufficient volume of microalgae solution was prepared in three stages of volume enhancement. At the first stage, 50 mL of algal stock solution was inoculated into a 500 mL BG11 medium with the following composition (mg.L^−1^): FeEDTA 1; MgSO_4_.7H_2_O 75; CaCl_2_.2H_2_O 36; FeCl_3_ 4; K_2_HPO_4_ 31; NaNO_3_ 1500; C_6_H_8_O_7_ (citric acid) 6; C_6_H_8_FeNO_7_ (ferric ammonium citrate) 6; Metal solution 1 mL.L^−1^. The composition of metal solution was as follows (mg.L^−1^): ZnSO_4_.7H_2_O 222; Na_2_MoO_4_.2H_2_O 39; CuSO_4_.5H_2_O 79; MnCl_2_.4H_2_O 1810; H_3_BO_3_ 2860; Co(NO_3_)_2_.6H_2_O 4.9. The culture was aerated by sparging air enriched with approximately 6% carbon dioxide at a rate of 1 vvm. The temperature was maintained at 27 °C, and fluorescent lamps were used to deliver a 6000 lx light intensity during a 16:8 h (light: dark) photoperiod. The microalgae culture was grown until the mid-logarithmic phase (day 3), and then it was used as the 3 L (6 × 500 mL) BG11 medium inoculum for further cultivation under the condition mentioned above. The third phase was conducted using a 60 L flat-plate photobioreactor (100 cm × 60 cm × 10 cm) with a working volume of 50 L to obtain adequate algal biomass for the study. The white LED lighting system of the photobioreactor was set to provide 12000 lx of illumination in a 16:8 h (light: dark) photoperiod. The algal solution derived from the previous step was used to inoculate the flat-plate photobioreactor with a ratio of 6% (vol/vol). An air stream containing 6% carbon dioxide was continuously bubbled into the medium. The temperature of the culture was maintained at 27 °C. Sodium bicarbonate was added in a fed-batch manner as an additional inorganic carbon source in the growth phase resulting in 2 g.L^−1^ of sodium bicarbonate in the medium (35 g on day 1, 30 g on day 2, 20 g on day 3, and 15 g on day 4). For harvesting at the end of cultivation, the pH of the culture was adjusted to 12 using sodium hydroxide, and then upon sedimentation, the concentrated algal solution was collected.

### Cell disruption

Break down of the cell wall was carried out using a combination of four disruption techniques: drying, grinding, organic solvent treatment, and ultrasonication. First, the concentrated algal solution was divided into two aliquots and dried using two methods: (a) oven-drying and (b) freeze-drying. For oven drying, the algal slurry was placed in an oven (WiseOven, South Korea) at 45 °C for 48 h. Freeze drying was performed at – 50 ºC for 72 h (FD-10 V, Pishtaz Engineering, Iran). Second, the dried microalgae biomass was ground by an electric mortar (VI-907, Iran) for 3 min. Third, 19.4 mL chloroform, and 10.2 mL methanol were added to 1 g of algal powder (ratio of 1.9/1) and mixed for a few minutes. Fourth, ultrasonication of the algal solution was conducted using an ultrasonic bath (WiseClean, WUC-A03H, South Korea) at 50 kHz for 20 min in two 10-min cycles and 1 min of resting time in order to prevent overheating the sample. Then, the mixture was stirred for 2 h at 760 rpm using a magnetic stirrer at room temperature (25 °C). The final solution was used for further extraction of bioproducts.

### TEM imaging

Both fresh and disrupted algal cells were subject to TEM imaging. The fresh samples were directly obtained from algal solution, while disrupted cells were obtained from treated solution. The samples were prepared by fixing in 2.5% glutaraldehyde in 0.1 M sodium cacodylate buffer solution for 2 h. Next, fixation of the slides was done in 1% osmium tetroxide for 2 h, followed by rinsing in 0.1 M sodium cacodylate buffer. The samples were then immersed in propylene oxide and embedded in Embed-812 epoxy resin. The cell Sections (70–85 nm) were obtained using a Leica EM UC6 ultramicrotome. Next, staining was carried out with uranyl acetate 1% and lead citrate and examined with a ZEISS Leo 912 AB transmission electron microscope.

### Lipid extraction

For lipid extraction, 3.4 mL of water was added to the treated solution and mixed for 1 min. The final mixture was centrifuged at 4 °C (4400 rpm, 10 min), and the chloroform layer was collected. The residual biomass was extracted a second time by contacting with 9.7 mL of chloroform. Both chloroform extracts were combined. The total lipid of the dried algae sample was determined gravimetrically by evaporating the chloroform solvent under nitrogen flow until constant weight. The lipid yield was calculated as the ratio of the mass of extracted lipid (g) to the mass of sample dried biomass (g).

### Carbohydrate extraction

The Carbohydrate content of algal biomass can be structural and soluble (non-structural). The structural carbohydrates are bound in the biomass matrix, while non-structural carbohydrates are soluble and can be extracted through washing steps. For the determination of soluble carbohydrate content, the supernatant of the lipid extraction stage was analyzed. Due to the presence of chlorophyll in the supernatant, it was decolorized using activated carbon, and then chlorophyll-free samples were used for analysis. The soluble carbohydrate concentration was determined using the phenol–sulfuric acid method (DuBois et al. 1956). Briefly, 0.5 mL of supernatant was reacted with 0.5 mL of phenol (5%) and 2.5 mL of concentrated sulfuric acid (> 96%) to create a characteristic yellow-orange color. The mixture was incubated for 20 min at room temperature, then the absorbance was measured (490 nm) using a spectrophotometer (UV–VIS 2100, UNICO, USA) and compared to a standard curve based on glucose as follows: C_c_ = 113.08 × OD_490_, where C_c_ is soluble carbohydrate concentration in µg.mL^−1^. Based on 13.6 mL of supernatant (10.2 mL methanol + 3.4 mL water), soluble carbohydrate content was calculated as the ratio of the total mass of soluble carbohydrate (g) to the mass of sample dried biomass (g).

To determine the structural carbohydrate content of the sample, acidic hydrolysis was performed. The cellular residues obtained from the lipid extraction step were dried in the oven and used for saccharification based on a method proposed by the National Renewable Energy Laboratory, USA (Sluiter et al. 2008). In summary, this method consists of two steps: In the first step, the initial hydrolysis, 3 mL of 72% sulfuric acid was added to 0.3 g of microalgae defatted-biomass and allowed to incubate at 30 °C for 1 h. In the second step, secondary hydrolysis, 84 mL of distilled water was added to the sample of the previous step (diluted to 4% acid concentration) and incubated in an autoclave at 121 °C for 1 h. After cooling to room temperature, the sample was centrifuged (4500 rpm, 10 min), and the carbohydrate content of the supernatant was determined using the phenol–sulfuric acid method, as described in the previous paragraph.

### Protein extraction

For the determination of hydrosoluble protein content, the supernatant of the lipid extraction stage was analyzed. Lowry kit (Tali protein assay kit, Taligene, Iran) was used for hydrosoluble protein analysis of 0.5 mL of supernatant according to the provided protocol. A calibration curve was prepared using a BSA concentration range from 0 to 160 μg.mL^−1^. The blue color solution absorbance was measured at 650 nm against a blank containing all the reagents excluding the extract and compared to a standard curve based on BSA as follows: C_c_ = 68.263 × OD_650_, where C_c_ is protein concentration in µg.mL^−1^. Based on 13.6 ml of supernatant, protein content was calculated as the ratio of the total mass of protein (g) to the mass of sample dried biomass (g).

The membrane protein content of the algal sample was determined based on the evaluation of total nitrogen content. The total nitrogen was evaluated using CHNS analysis (Thermo Finnigan, FLASH EA 1112 SERIES) and then the protein content was determined using Eq. (1) (Safi et al. 2013):

1 $${\text{C}}_{{\text{p}}} = {\text{ C}}_{{\text{N}}} \times {\text{ NTP}}$$

where NTP is the nitrogen-to-protein conversion factor. The algal sample (2 mg) was placed in a thin capsule and heated at 925 °C, using pure helium as the carrier gas and pure oxygen as the combustion gas, and the nitrogen concentration was measured in the flue gas.

### Ash content

To determine ash content, 0.5 g of dry biomass (freeze/oven-dried) was poured into a pre-weighted aluminum container and placed in the oven at 105 °C for 2 h. Next, the container was weighed and placed in a furnace at 575 ± 25 °C for 2 h. Then, the container was placed in a desiccator to cool down to room temperature, and the net weight of ash was determined immediately.

### Statistical analysis

All extraction experiments were performed twice, and the reported values are the mean of the experimental results with a margin of error at the confidence level of 95%. For the calculated results, the margins of error were calculated according to the significant figures rule. A P-value criterion with a confidence level of 0.95 was used to investigate the significance of the difference between the results. The Z-score parameter was used to calculate P-values.

## Results

Sequential extraction of bioproducts (lipid, carbohydrate, and protein) from the same sample was performed on three algal species. Conventionally biochemical assays are carried out on separate samples, and the content of each component is reported independently. Sequential extraction of bioproducts from the same sample is an approach that results in more realistic contents of bioproducts since the effect of extracting one component on the other components is already implemented in the analysis. This approach is also more practical than single-product extraction methods in real-world applications because downstream treatments always take place on the same amount of algal biomass. Thus, the biochemical contents reported in this study are based on this approach, and extraction of lipid, carbohydrate, and protein are applied to the same algal sample. Following this approach, it is hypothesized that after successful cell disruption, and extraction of biomacromolecules into the organic-aqueous medium, lipids are extracted into the organic phase, both soluble proteins and non-structural carbohydrates are dissolved into the aqueous phase, while structural carbohydrates and membrane-bound proteins remain in residual biomass. Thereby, samples are collected from the appropriate phase and analyzed.

### Cell disruption and TEM imaging

**Fig. 1:**
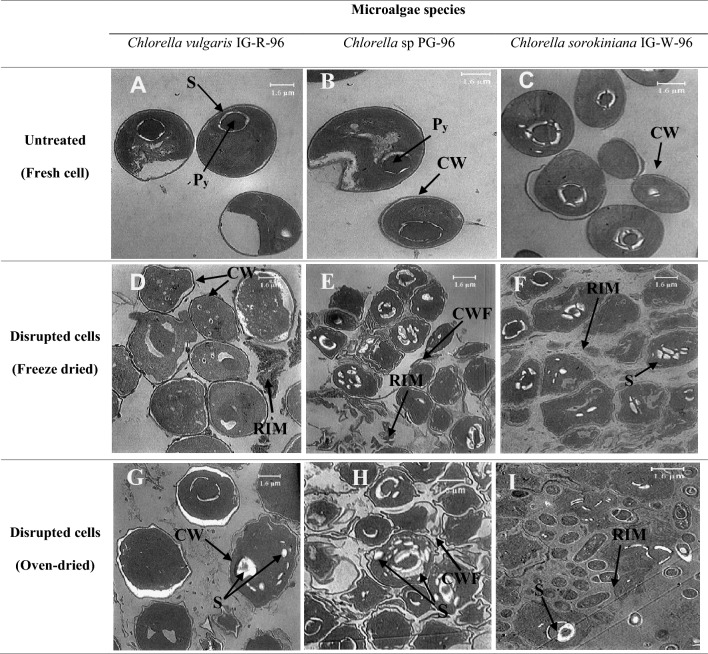
TEM imaging of untreated (**A**–**C)**, freeze-dried (**D**–**F)** and oven-dried (**G**–**I)** cells of *C. vulgaris* IG-R-96, *C. sorokiniana* IG-W-96 and *Chlorella *sp PG-96 samples at 1250 × magnification. Letters that indicate components of cell: (S) starch granule, (Py) Pyrenoid, (CW) cell wall, (CWF) cell wall fragments, (RIM) released intracellular material

Effective bioproduct extraction highly depends on the success of cell disruption. Published literature has shown that combining cell wall disruption techniques is more efficient than a single technique (El-Sheekh and Hamouda 2016; Zhang et al. 2018). Thus, the combination of drying, grinding, organic solvent treatment, and ultra-sonication were used to disrupt the cell walls of three studied microalgal cells effectively. The degree of cell disruption was qualitatively evaluated through TEM imaging. Figure 1 shows the TEM images of three algal cells before and after-disruption treatments under two drying methods, i.e., freeze-drying and oven-drying. As seen in this figure, untreated cells have spherical shapes and intact walls. The cell components, such as pyrenoid and starch granules, are visible within the cell boundaries (Fig. 1A–C). In contrast, after disruption most algal cells underwent deformation, some cell walls were broken, and deterioration of cell integrity caused a release of intracellular material (Fig. 1D–I). In *Chlorella vulgaris* IG-R-96 (Fig. 1D, G), cells are more resistant to disruption than the two other species. Cell wall shrinkage is visible in freeze-dried and oven-dried samples, while released intracellular material (RIM) indicates that some of the freeze-dried cells were torn apart. In *Chlorella* sp PG-96 (Fig. 1E, H), the degree of cell disruption is qualitatively satisfactory, and biomacromolecules are released into the medium. Cell wall fragments are visible specifically in the freeze-dried sample, and cell integrity is somewhat lost. In *Chlorella Sorokiniana* IG-W-96, disruption methodology is the most effective, and cell wall fractions are abundant (Fig. 1F, I). Intracellular materials are released into the medium, and carbohydrate granules are broken apart. Almost no integrated cell structure is visible, and no cell boundary can be identified.

Generally, *Chlorella* genus, including *C. vulgaris* and *C. sorokiniana* are known to have cell walls of polymeric structures with the thickness of 17–20 nm and 22 nm, respectively (Grossmann et al. 2018), that contribute to the rigidity of cell walls. In *C. vulgaris* and *C. sorokiniana,* glucosamine, as the main constituent of these structures, strengthens the rigidity of the cell wall and consequently makes the cell wall rupture more difficult (Phong et al. 2018). Nevertheless, the composition of the cell wall of *Chlorella* genus is species-specific. Mainly, the *Chlorella* cell wall composition consists of a two-layer structure rich in cellulose, hemicellulose, and pectin. The outer layer comprises algaenan, a resistant aliphatic biopolymer containing long-chain saturated hydrocarbons cross-linked by functional groups such as esters. This complicated structure is the main factor in the strength of the algal cell wall (Alhattab et al. 2019).

### Effect of drying on extraction yields of bioproducts

#### Lipid extraction yield

Lipid is one of the macromolecules recovered from algal biomass through sequential extraction. This type of biomacromolecule is obtained from the organic phase of homogenized biomass after cell wall disruption treatments. Table 1 indicates the lipid yields of three algal species under freeze-drying and oven-drying treatments. As seen in Table 1, the lipid yields of freeze-dried samples were higher than those obtained from oven-dried samples, so freeze-drying significantly increased lipid yields for *Chlorella sorokiniana* IG-W-96, *Chlorella* sp. PG-96, and *Chlorella vulgaris* IG-R-96, approximately 7%, 17%, and 5.5% compared to oven-drying; respectively. Calculating p-values between lipid yields in freeze-dried and oven-dried samples in each species (*C. sorokiniana* IG-W-96: 0.02; *Chlorella* sp. PG-96: 1.2 × 10^–6^; *C. vulgaris* IG-R-96: 0.12) showed that the difference is significant for *Chlorella sorokiniana* IG-W-96, and *Chlorella* sp. PG-96, while it is not significant for *Chlorella vulgaris* IG-R-96*.* This observation suggests that freeze-drying is a more effective technique in cell rupture than oven-drying. TEM images also show that freeze-dried cells have undergone more destruction when subject to disruption treatments than oven-dried cells, hence the damage to the cell structure and release of cell material is more visible in freeze-dried biomass than in oven-dried biomass. It is reported that drying algal cells in high temperatures, as it occurs in oven drying, causes hardening of cell cases demanding higher energy input for cell break and biochemical release (Ansari et al. 2018). Moreover, prolonged oven-drying would increase lipid oxidation, resulting in a lower lipid content than freeze-drying. In confirmation, *Chlorella sorokiniana* IG-W-96 strain and *Chlorella vulgaris* IG-R-96 demonstrated the highest and lowest lipid yield, respectively, in both freeze-dried and oven-dried samples. This indicates that the cell wall of *Chlorella vulgaris* IG-R-96 appeared to be more resistant to rupture, confirming the critical role of cell wall rigidity in lipid extraction. Previous studies have reported that the lipid extraction yield of *Chlorella sorokiniana* ranges from 8.8% to 19.8% (w/w) (Gupta et al. 2017). Also, it has been reported that despite rigidity, ultrasonication can lead to better penetration of the solvent into the rigid cell matrix of *Chlorella vulgaris* and ease the extraction process (Naveena et al. 2015). Another study also observed that ultrasonication during disruption could increase the extraction yield of lipid from *Chlorella vulgaris* microalgae biomass up to 52.5% w/w (Chen et al. 2013), which is somewhat unrealistic compared to peer studies.

**Table 1:** Lipid yields of three studied microalgae under two different drying methods

Drying methods	Lipid yield (% DCW)		
	*Chlorella vulgaris* IG-R-96	*Chlorella* sp. PG-96	*Chlorella sorokiniana* IG-W-96
Freeze-dried	9.8 ± 0.5	13.9 ± 0.6	15.4 ± 0.6
Oven-dried	9.3 ± 0.7	11.8 ± 0.6	14.4 ± 0.8

The data points in each column are the average values of replicated experiments with margin of error at confidence level of 95%

#### Carbohydrate extraction yield

**Fig. 2:**
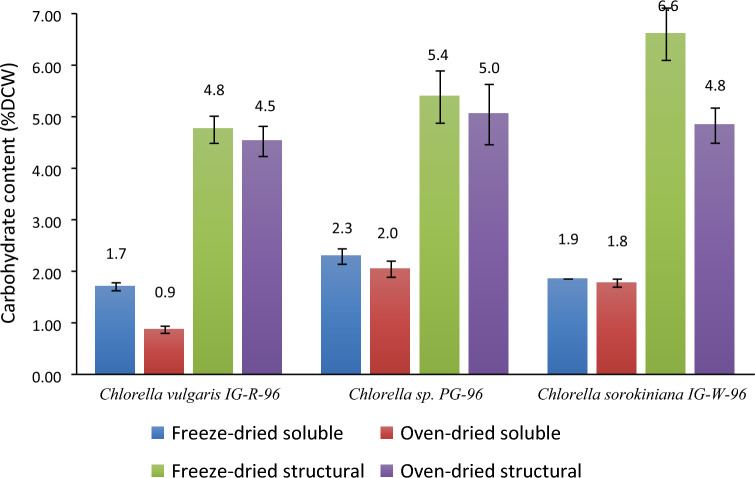
Extraction yields of carbohydrates (soluble and structural) in three algal species using two drying methods. Error bars indicate the mean of the experimental values with margin of error at the confidence level of 95%

The soluble carbohydrate content of algal samples was recovered from the supernatant of the extraction medium. Figure 2 indicates the carbohydrate yield of three freeze-dried and oven-dried species. As seen in this figure, the soluble carbohydrate yield of freeze-dried samples was significantly higher than those obtained from oven-dried samples (p-values: *C. sorokiniana* IG-W-96: 0.04; *Chlorella* sp. PG-96: 0.01; *C. vulgaris* IG-R-96: ~ 0), suggesting that freeze-drying is more effective than oven drying in biomacromolecule extraction including soluble carbohydrate. The highest soluble carbohydrate yield of 2.3 ± 0.2% was observed in the freeze-dried sample of *Chlorella* sp. PG-96 microalgae biomass, while the lowest yield of 0.9 ± 0.1% was obtained from oven-dried *Chlorella vulgaris* IG-R-96 sample biomass. Figure 2 also shows that the soluble carbohydrate yield of the freeze-dried *Chlorella vulgaris* IG-R-96 sample (1.7 ± 0.1%) is approximately double compared of the oven-dried sample (0.9 ± 0.1%). These observations indicate that in algal species with rigid cell walls, e.g., *C. vulgaris*, the type of drying makes a difference, so that freeze-drying is more efficient than oven drying, However, in algal species with less rigid cell walls, there is a slight variation between freeze-drying and oven-drying.

In microalgae cells, soluble carbohydrates are primarily found in the form of starch, cellulose, and other polysaccharides (Chen et al. 2013). Through sonication, chain scission is facilitated in polysaccharides; thus, a small fraction of polysaccharides is degraded into their building blocks, such as glucose (Yan et al. 2016). In many studies regarding the saccharification of polysaccharides, ultrasound is typically used as a pretreatment. Its effects are limited, which may justify the small amount of carbohydrates in the aqueous phase of the extraction. Furthermore, the result of freeze-drying on cell wall breakage could be seen from the significant increase in soluble carbohydrate yield of *Chlorella vulgaris*, as the high sugar concentration indicates efficient breakdown and degradation of cell wall components. Given this, and as demonstrated by TEM images, the difference in soluble carbohydrate yield could be a definitive indicator of how cells are disrupted.

Structural carbohydrates include compounds such as cellulose, pectin, and hemicellulose found in the structure of cell or organelles membranes. These carbohydrates are not directly obtained by disrupting cells and require acidic hydrolysis. For this purpose, the microalgae biomass was dried after lipid extraction and hydrolyzed using concentrated sulfuric acid. Figure 2 shows the structural carbohydrate yield of the three microalgae species. The maximum and minimum carbohydrate yields were found in the freeze-dried *Chlorella sorokiniana* IG-W-96 (6.6 ± 0.5%) and oven-dried *Chlorella vulgaris* IG-R-96 (4.5 ± 0.3%)*;* respectively. The difference between structural carbohydrate yields in drying methods was solely significant in *C. sorokiniana* IG-W-96 (p-value = 7.4 × 10^–9^). In the other two species, the differences were not significant (*Chlorella* sp. PG-96: p-value = 0.19; *C. vulgaris* IG-R-96: p-value = 0.13). The lower yield of structural carbohydrates in *Chlorella vulgaris* IG-R-96 compared to two other species may be attributed to the rigid cell wall of this species, as observed in TEM images of disrupted cells.

#### Protein extraction yield

Proteins are one of the main constituents of microalgal cells. Protein synthesis in living cells is the most complicated mechanism. Conventional cell disruption techniques were used to release the protein into the aqueous phase using the centrifugation step. The recovery of hydro-soluble proteins from the supernatant was performed after solvent extraction. The fraction of hydro-soluble proteins released into the aqueous phase using two different drying methods is less than 0.5% for all three strains. The negligible amount of hydro-soluble protein content of the treated samples might be due to the degradation and/or denaturation of some proteins due to disruption of the cell wall in sequential extraction. Previous studies noticed that some proteins were denatured during the extraction of lipids with the mixture of chloroform and methanol solvents in *Nannochloropsis* microalgae biomass (Gerde et al. 2013). Due to the existence of yet undetermined compounds in the structure of cell walls, and various disruption propensities of different microalgae species, subjecting them to the same treatment might result in variations in the protein yield from three *Chlorella* species (Phong et al. 2018). Therefore, to increase the protein extraction yield, more effective and diverse cell wall disruption techniques are required (Gerde et al. 2013). Furthermore, two different drying methods imposed various effects on protein extraction yields of the three species.*,* When treated by freeze-drying, *Chlorella sorokiniana* IG-W-96 and *Chlorella* sp. released lower amounts of hydro-soluble proteins, possibly due to the inactivation of proteins through disulfide exchange and other reactions in the freeze-drying process (Roy and Gupta 2004), while mild heat in the oven did not impair the stability of proteins.

**Fig. 3:**
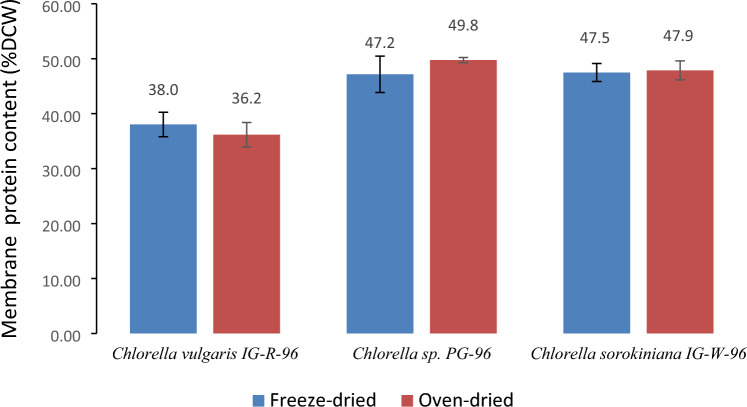
Membrane protein extraction yields of three studied microalgae under two different drying methods. The data points in each column are the average values of replicated experiments with margin of error at confidence level of 95%

Because, intracellular proteins are distributed among cytoplasm and cell membranes, not all proteins are found in the aqueous phase; instead, most proteins are known to be membrane-bound proteins. As a result, the extraction of hydrosoluble proteins does not represent the total protein content of the cell. For this purpose, CHNS (Sect.  2.8) analysis was performed to evaluate the total protein yield of algal samples. Using the nitrogen to protein (NTP) conversion factor of 6.35, nitrogen yield can be converted to protein yield (Safi et al. 2013). However, the traditional NTP conversion factor of 6.35 is not recommended for all microalgae species; because a significant amount of non-protein nitrogenous substances contained in pigments, nucleic acids, and inorganic components are accounted for. As such, the specific NTP conversion factor of 4.67 is proposed for *C. vulgaris* microalgae, and the NTP conversion factor of 4.78 is used in calculations for *Chlorella* sp. and *Chlorella sorokiniana* microalgae (Phong et al. 2017). Figure 3 indicates membrane protein extraction yields for the three species based on the nitrogen content of each sample. The results show that the highest and lowest percentages of membrane proteins belong to oven-dried *Chlorella* sp. PG-96 (49.8 ± 0.3) and oven-dried *C. vulgaris* IG-R-96 (36.2 ± 2.6), respectively. Nevertheless, the differences between membrane protein yields of oven-dried and freeze-dried samples in three species are not significant (p-values: *C. sorokiniana* IG-W-96: 0.38; *Chlorella* sp. PG-96: 0.06; *C. vulgaris* IG-R-96: 0.12), suggesting that the membrane protein yield is independent of the drying method and cell disruption treatment.

#### Ash

For mass balance, the ash contents of the three microalgae species were also determined at two drying methods, and the results are reported in Table 2. As seen in this table, the highest amount of ash was obtained from freeze-dried *Chlorella sorokiniana* IG-W-96, even though there is no significant difference between freeze-dried and oven-dried results in this species (p-value = 0.08). *Chlorella* sp PG-96 produced the lowest ash content either in freeze-dried or oven-dried samples (p-value = 0.5). In the case of *Chlorella vulgaris* IG-R-96 significant difference (p-value = 0.01) was observed in ash content between oven-dried and freeze-dried samples. The result is in agreement with the findings of a similar study, which indicated that freeze-dried biomass accumulates a greater level of ash than other drying methods (Shekarabi et al. 2019).

**Table 2:** Ash content of three microalgae species after cell disruption and biochemical extraction

Microalgae	Ash content (%DCW)	
	Oven-dried	Freeze-dried
*Chlorella vulgaris* IG-R-96	20.5 ± 2.1	23.1 ± 1.5
*Chlorella* sp. PG-96	20.5 ± 2.1	20.5 ± 1.4
*Chlorella sorokiniana* IG-W-96	22.1 ± 1.3	24.0 ± 1.4

The data points in each column are the average values of replicated experiments with margin of error at confidence level of 95%

### Sequential extraction versus independent assays

Although in both sequential extraction and independent assays similar extraction techniques are applied, the independent assays are dealing with fresh (separate) samples while sequential extraction is dealing with the same sample. Regarding this difference in the extraction of value-added bioproducts and commodities, it is essential to evaluate the maximum economic potential of sustainable and renewable algal feedstock and its extraction methodology (Wychen et al. 2021). This potential may be realized through accurate mass closure of algal samples to achieve maximum potential. Table 3 presents ash-free and ash-plus total biochemicals recovered through sequential extraction versus independent assays to evaluate the potential disruption and fractionation techniques utilized in this study. The ash-free figures reveal that the sequential extraction was substantially successful compared to independent assays in recovering as much as cellular products. The overall yield is higher than that in independent assays. For example, when independent essays were applied on *Chlorella sorokiniana* IG-W-96, total ash-free bioproducts yield obtained as much as 46.2 ± 1.9% of DCW (protein 24.8 ± 1.0%, carbohydrate 11.2 ± 0.8%, lipid 10.2 ± 0.2%) which is lower than 68.9 ± 1.5% achieved through sequential extraction. This is also the case in two other studied algal species as observed in Table 3. In another study, which employed independent assays for each biomacromolecule, they recovered less ash-free total bioproducts of 64.5% of DCW of *Chlorella sorokiniana* compared to this study (protein 37.8%, ydrate 17.8%, lipid 8.9%) (Sayedin et al. 2020).

**Table 3:** Total biochemical extraction yields for three microalgae species using sequential extraction and independent assays

Microalgae	Sequential extraction (%DCW)				independent assays (%DCW)	
	Oven-dried		Freeze-dried		Oven-dried	Freeze-dried
	Ash-free	Ash-plus	Ash-free	Ash-plus	Ash-free	Ash-free
*Chlorella vulgaris* IG-R-96	50.9 ± 2.7	71.4 ± 4.8	54.5 ± 2.7	77.6 ± 4.2	36.2 ± 1.9	38.4 ± 1.5
*Chlorella* sp. PG-96	68.9 ± 0.9	89.5 ± 3.0	68.8 ± 2.5	89.3 ± 3.9	47.6 ± 2.1	48.2 ± 1.7
*Chlorella sorokiniana* IG-W-96	68.9 ± 1.5	91.0 ± 2.8	71.4 ± 1.4	95.4 ± 2.8	46.2 ± 1.9	49.4 ± 1.5

For calculated results in this table, the margins of error were calculated according to the significant figures rule

## Discussion

Proper disruption of microalgae cell walls lets biochemicals fully release into the medium, thus improving the extraction yields (Sierra et al. 2017). Proper disruption may involve a combination of various methods to affect different parts of the cell, including cell and organelle membranes in eukaryotes. The 4-step disruption strategy utilized in this study, i.e., drying, grinding, organic solvent treatment, and ultrasonication, was effective in the sense that biomass drying, regardless of drying type, increases cell wall permeability, and the following grinding breaks the fragile and rigid wall of the cell. The extracting solvents penetrate the cell and dissolve available macromolecules released from the cellular locations. Finally, the cavitation caused by associated ultrasonication increases the solubility of macromolecules into the solvents and completes the disintegration of cell boundaries. The completeness of this operation indicates the success of cell disruption and consequently improves bioproducts’ sequential extraction yields.

The ash-plus figures (Table 3) indicate that the overall mass balance is species-dependent in terms of cell wall structure and toughness. *Chlorella vulgaris* IG-R-96 with the most robust cell wall underwent lower ash-plus extraction (71.4 ± 4.8%), while *Chlorella sorokiniana* IG-W-96 with a less intense cell wall demonstrated the highest extraction (95.4 ± 2.8%) from the dried cell. While most of the difference may be explained by the variation in biochemical accumulation characteristics of the microalgae strain, it may be attributed to the cumulative effects of both rigid cell wall structure and the tendency of *Chlorella vulgaris* to produce low levels of lipid/soluble protein/soluble carbohydrate under the cultivation conditions.

Table 3 also shows that drying type can make a difference, so freeze-drying is significantly more effective than oven-drying for species such as *Chlorella vulgaris* IG-R-96 (8.6% increase ash-plus, p-value = 0.003). From the biorefinery standpoint, the non-significant performance of freeze-drying for *Chlorella sorokiniana* IG-W-96 (p-value = 0.085) and *Chlorella* sp. PG-96 (p-value = 0.466) is a potential economic advantage. In view of the relatively high operating costs of freeze-drying and the better potential of oven-drying for field applications, it might be preferable to use oven-drying as a reasonable alternative in such cases.

## Data Availability

The data used in this study is available upon reasonable request onding author.
